# Blockade of nicotine sensitization by methanol extracts of *Glycyrrhizae* radix mediated via antagonism of accumbal oxidative stress

**DOI:** 10.1186/s12906-017-1999-2

**Published:** 2017-11-16

**Authors:** Zheng Lin Zhao, Sang Chan Kim, Hong Feng Liu, Yi Yan Wu, Li Bo Li, Yu Hua Wang, Yu Jiao, Yu Fan, Chul Won Lee, Bong Hyeo Lee, Il Je Cho, Chae Ha Yang, Rong Jie Zhao

**Affiliations:** 1 School of Mental Health, Qiqihar Medical University, 333 Bukuibei Street, Jianhua District, Qiqihar, 161006 China; 2 College of Korean Medicine, Daegu Haany University, Gyeongsan, 38610 Republic of Korea; 3 Department of Pharmacology, Mudanjiang Medical University, Mudanjiang, 157011 China

**Keywords:** Nicotine, Sensitization, *Glycyrrhizae* radix, Dopamine, Nucleus accumbens, Oxidative stress

## Abstract

**Background:**

We previously reported that a methanol extract of *Glycyrrhizae* radix (MEGR) blocked methamphetamine-induced locomotor sensitization and conditioned place preference in rats. In the present study, the effects of MEGR on repeated nicotine-induced locomotor sensitization and enhanced extracellular dopamine (DA) release in the nucleus accumbens (Nacc) were evaluated.

**Methods:**

Male Sprague–Dawley rats received repeated administrations of nicotine (0.4 mg/kg, subcutaneous) or saline twice a day for 7 d and were challenged with nicotine 4 d after the last daily dosing. During the 4-d withdrawal period, the rats were treated once a day with MEGR (60 or 180 mg/kg/d). Extracellular DA levels were measured by in vivo microdialysis, the malondialdehyde levels and the activities of superoxide dismutase and catalase in the Nacc were biochemically evaluated, and the expression of antioxidant proteins was confirmed by Western blot assays. All data were assessed with analysis of variance tests followed by post-hoc comparison tests and *p* values <0.05 were considered statistically significant.

**Results:**

The expression of repeated nicotine-induced locomotor sensitization was dose-dependently attenuated by MEGR, and 180 mg/kg/d MEGR significantly inhibited augmented accumbal DA release induced by a direct local challenge of nicotine. Moreover, 180 mg/kg/d MEGR reversed increases in malondialdehyde production, decreases in superoxide dismutase and catalase activities, and the reduced expression of nuclear factor erythroid 2–related factor 2 and heme oxygenase 1 in the nicotine-sensitized Nacc.

**Conclusions:**

These results suggest that MEGR inhibited nicotine-induced locomotion and dopaminergic sensitization via antioxidant action.

## Background

It is now known that cigarette smoking can greatly endanger public health worldwide and that nicotine dependence is likely the prime culprit associated with the failure to quit smoking [1]. However, other than nicotine replacement therapy, which has limited effectiveness, there are no available pharmacotherapies that effectively aid quitting [2]. Although nicotine has a relatively lower potency than most opiates and psychostimulants, its rewarding effect is a key motivational factor that sustains nicotine dependence [3]. In rodent studies, the rewarding effects of nicotine typically manifest as behavioral sensitization such as enhancement in locomotor activity that occurs after repeated nicotine treatment (RNT) [4]. Additionally, this sensitization is positively associated with other addiction-related behaviors, such as nicotine seeking and self-administration.

Similar to other addictive drugs, nicotine induces increased levels of accumbal dopamine (DA) release; these changes are neurochemically responsible for the rewarding effects of the drug [5]. Furthermore, RNT leads to a sensitized dopaminergic state such that a challenge dose of nicotine in a subject pre-exposed to nicotine produces a significant augmentation in accumbal DA release compared with a subject naive to nicotine [6]. This process is thought to be the neurobiological underpinning of behavioral sensitization.

To date, the exact biochemical mechanisms underlying the sensitization of the dopaminergic response after repeated treatment with drugs have yet to be fully elucidated. However, an increasing amount of evidence points to the critical role of reactive oxygen species (ROS) in this regard [7]. There are increased ROS levels in brain regions related to reward after repeated exposure to a variety of abused drugs, including nicotine [8]. Additionally, TEMPOL, which is a ROS scavenger, attenuates the promotion of cocaine and methamphetamine self-administration by preventing the sensitization of DA release in the nucleus accumbens (Nacc) [9, 10]. Further, abolishment of oxidative stress in the Nacc and prefrontal cortex can block repeated cocaine-induced rat locomotor sensitization [11]. Therefore, it is possible that the increase in accumbal oxidative stress induced by repeated administration of drugs of abuse might be a promising target for the treatment of addiction.

The radix of *Glycyrrhizae uralensis* [*Glycyrrhizae* radix (*G.* radix)] has traditionally been used in Oriental medicine for detoxification, the treatment of various injuries, and swelling, due to its ability to replenish and invigorate deficiencies in “Qi and Blood” [12]. *G.* radix contains flavonoids and different types of pentacyclic triterpene saponin, a group which includes liquiritigenin, liquiritin, isoliquiritigenin, glycyrrhizin, and glycyrrhizic acid [13]. Historically, *G.* radix has received pharmacological interest due to its anti-oxidant, anti-inflammatory, antitumor, and antitussive actions [14, 15]. However, over the past decade, several studies have revealed *G.* radix has neuropharmacological properties such as neuroprotective, antidepressant, and anxiolytic effects [16–18]. In previous studies, we found extracts from *G.* radix suppressed acute cocaine- and methamphetamine- induced increases in accumbal DA levels [19] and prevented acute methamphetamine-induced hyperlocomotion in rats [20], indicating inhibitory effects of *G.* radix against pharmacological actions of psychostimulants. Moreover, in other studies, we also demonstrated *G.* radix improved the behavioral and neurochemical abnormalities caused by repeated psychostimulant treatment such that extracts from *G.* radix attenuated methamphetamine-induced locomotor sensitization and conditioned place preference [21] and isoliquiritigenin ameliorated repeated methamphetamine-induced loss of striatal dopamine transporter densities and tyrosine hydroxylase activities [22]. Since nicotine belongs to the category of psychostimulants and shares common neurochemical and behavioral traits with the typical psychostimulants when given repeatedly [23], the results of these studies collectively raise the possibility that *G.* radix can prevent the occurrence of aberrant neurochemical and behavioral changes by RNT.

In the meantime, in the above mentioned studies, we have shown that *G.* radix exerts its neuropharmacological actions via glutamatergic and gamma-aminobutyric acid (GABA)ergic receptors [19, 21], which is also supported by the study done by others [24]. However, since the primary pharmacological property of *G.* radix is its antioxidant capabilities, and as reported by Zeng et al. that isoliquiritigenin alleviated intracerebral hemorrhage-induced brain injury in rats via suppressing excessive ROS production [25], it is possible that the neuroprotective effects of *G.* radix against repeated psychostimulant treatment are mediated, at least in part, via its antioxidant activities.

Therefore, in the present study, we aimed to evaluate the potential therapeutic effects of *G.* radix on RNT-induced locomotor and accumbal dopaminergic sensitization in rats and to further determine whether these effects are associated with its antioxidant actions.

## Methods

### Preparation of an extract of *G.* Radix

The methanol extract from G. radix (MEGR) was prepared as previously described [21]. Briefly, *G.* radix was obtained from Dae-Won pharmacy (Daegu, Republic of Korea), identified by Professor Sang Chan Kim (College of Oriental Medicine, Daegu Haany University, Daegu, Republic of Korea) and ground into fine powder. The powdered *G.* radix was extracted in methanol for 48 h, filtered through a 0.22-μm filter (Nalgene; NY, USA), and then lyophilized in a vacuum evaporator. The amount of MEGR was estimated based on the dry weight of the lyophilized MEGR; the yield was 18.36%. Analysis with high-performance liquid chromatography (HPLC) has shown that MEGR contains glycyrrhizic acid, liquiritigenin, and isoliquiritigenin [21].

### Animals

Male Sprague–Dawley rats were obtained from the Laboratory Animal Center at Qiqihar Medical University (Qiqihar, China). The rats were 9 weeks old (280-300 g) at the beginning of the experiments, and housed in Plexiglas cages (3 rats per cage), provided with ad libitum food and water, maintained in a filtered pathogen-free air environment at a temperature of 21-23 °C and 50% relative humidity, and on a 12:12 h light/dark cycle. All animal procedures were performed in accordance with the National Institutes of Health Guide for the Care and Use of Laboratory Animals and were approved by the Animal Care and Use Committee of Qiqihar Medical University (Approval Number: QMU-AECC-2015-26).

### Locomotor activity test

Locomotor activity was assessed in a rectangular box (40 × 40 × 45 cm^3^) with a floor and walls that were made from clear Plexiglas painted black. The chamber was equipped with a video camera above the center of the floor, and all locomotor activity was monitored by a video tracking system using the Ethovision program (Noldus Information Technology BV, Wageningen, Netherlands).

### Nicotine-induced Locomotor sensitization and drug treatment

**Fig. 1:**
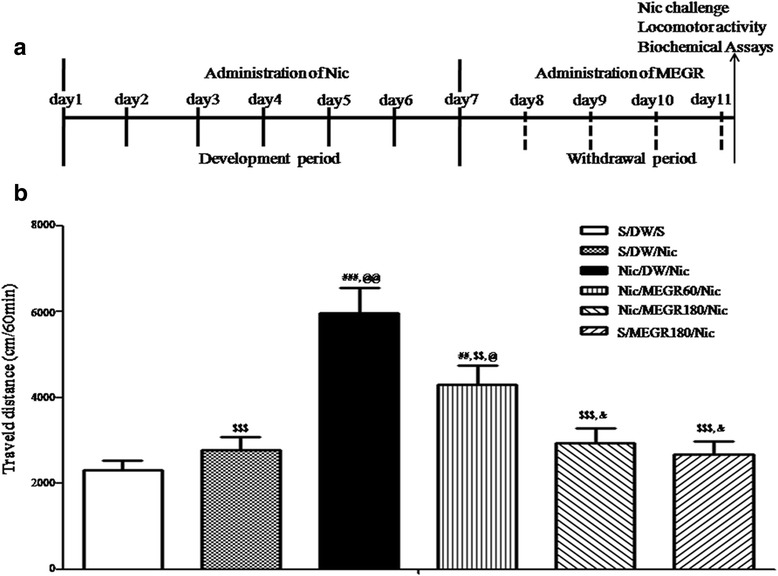
**a** Time schedule for nicotine-induced locomotor sensitization. Rats received nicotine (Nic) administrations (0.4 mg/kg s.c., twice a day) for 7 consecutive days, underwent 4 days of withdrawal, and then challenged with the same dose of Nic to induce locomotor sensitization; during the withdrawal period, the rats were orally administered MEGR (60 or 180 mg/kg/day, 4 times). **b** Effects of MEGR on Nic-induced locomotor sensitization in rats. Repeated Nic administrations induced a significant locomotor sensitization, which was inhibited by MEGR treatment. All data are expressed as means ± SEM (*n* = 7). S: saline, DW: distilled water, MEGR: methanol extract of *G.* radix, MEGR60: 60 mg/kg/day MEGR, MEGR180: 180 mg/kg/day. ^##^ *P* < 0.01, ^###^ *P* < 0.001 versus S/DW/S group; ^$$^ *P* < 0.01, ^$$$^ *P* < 0.001 versus Nic/DW/Nic group; ^@^ *P <* 0.05, ^@@^ *P* < 0.01, versus S/DW/Nic group; ^&^ *P* < 0.05 versus Nic/MEGR60/Nic group (one-way ANOVA followed by Newman-Keuls post-hoc test)

Forty two rats were given subcutaneous (s.c.) administration of either saline or 0.4 mg/kg of (−)-nicotine hydrogen tartrate (Sigma, St. Louis, MO, USA) dissolved in saline (pH 7.2; all doses expressed as free base) twice a day for 7 consecutive days in their home cages, randomly assigned to drug-treated groups (3 groups, *n* = 7) and vehicle-treated groups (3 groups, *n* = 7), and then underwent 4 d of withdrawal [6, 26]. During the withdrawal period, the rats were orally treated with either distilled water (DW) or MEGR (60 or 180 mg/kg/d, dissolved in DW) once a day for 4 d [20, 21]. Immediately after the final dose of DW or MEGR, the rats were adapted to the locomotor testing boxes for 60 min and then systemically challenged with either nicotine (0.4 mg/kg) or saline [26]. Following the challenge, the rats stayed in the boxes for an additional 60 min while locomotor activity was assessed (Fig. 1a). Immediately after the behavioral test, the rats were anesthetized with ether and decapitated, and the entire brain was removed and stored at −80 °C. Tissue samples from the Nacc shell (NaccSh; anterior–posterior: 1.7 mm, medial–lateral: 0.8 mm, and dorsal–ventral: −7.4 mm, based on the Paxinos and Watson rat brain atlas) [27] were punched from the stored brains for biochemical and Western blot analyses.

To investigate the effects of MEGR on sensitized accumbal DA release, another cohort of nicotine-withdrawn rats (submitted to the same treatment schedule described above) fitted with dialysis probes received a direct intra-NaccSh infusion of nicotine (3.0 mM) for 20 min [26] at 60 min after the fourth administration of MEGR. Subsequently, DA levels in the NaccSh were examined using in vivo microdialysis [26].

### Extracellular DA measurement

A microdialysis probe guide cannula (CMA11, Carnegie Medicin, Stockholm, Sweden) was implanted into the NaccSh using a stereotaxic instrument (Kopf Instruments, Tujunga, CA, USA) while the rats were under anesthesia (sodium pentobarbital, 50 mg/kg, intraperitoneally). After the surgery, the rats were individually housed, and antibiotic treatment (bacitracin ointment and penicillin) and acetaminophen were applied for 3 days to minimize infection and pain in the rats. Following 7 d of post-surgery recovery, the rats were subjected to the nicotine sensitization schedule. On the fourth day after the last daily injection, microdialysis probes (CMA11, cuprophane dialysis membrane, 6000 Da, 2-mm length) were inserted into the NaccSh via the guide cannula, and modified Ringer’s solution (MRS; 150 mM NaCl, 3.0 mM KCl, 1.4 mM CaCl_2_, and 0.8 mM MgCl_2_ in 10 mM phosphate buffer at pH 7.2) was perfused at a constant flow rate of 1.5 μL/min by using a microinjection pump in a microdialysis bowl. Microdialysis samples were collected at 20-min intervals in microcentrifuge tubes and three consecutive samples were assayed using an HPLC procedure with a coulometric detector (Coulochem II; ESA, Bedford, MA, USA) to measure basal DA levels prior to drug administration.

### Evaluation of oxidative stress parameters

The stored NaccSh tissue samples were sonicated (10%, *w*/*v*) in ice-cold 0.1 M phosphate buffer (pH 7.4), centrifuged at 700×*g* for 10 min at 4 °C, and the supernatants were collected to measure parameters of oxidative stress. Malondialdehyde (MDA) levels, which are an index of lipid peroxidation, were determined using a thiobarbituric acid method and an MDA kit (Jiancheng Bioengineering Institute, Nanjing, China) [14]. The activities of accumbal antioxidant enzymes, including superoxide dismutase (SOD) and catalase (CAT), were assayed spectrophotometrically using the respective kits from Jiancheng Bioengineering Institute. Protein concentrations in the brain homogenate samples were determined using the bicinchoninic acid protein assay.

### Western blot analysis

Cytosolic and nuclear proteins were extracted from the NaccSh samples for the detection of heme oxygenase 1 (HO-1) and nuclear factor erythroid 2–related factor 2 (Nrf2) using cytoplasmic and nuclear extraction reagents (Beyotime Biotech Inc., Nantong, China). Protein samples were separated using 12% sodium dodecyl sulfate polyacrylamide gel electrophoresis (SDS-PAGE) and then transferred to polyvinylidene difluoride (PVDF) membranes (Millipore, Bedford, MA, USA). The primary antibodies for the immunoblotting assays included a rabbit polyclonal antibody for Nrf2 (Abcam, Cambridge, UK) and a rabbit polyclonal antibody for HO-1 (Abcam); the secondary antibody was IRDye 800CW Goat anti-Rabbit IgG (Li-Cor Bioscience, Lincoln, NE, USA). Histone H3 (for nuclear proteins) and β-actin (for cytoplasmic proteins) were used as loading controls and detected with a rabbit polyclonal antibody (Abcam). The ODYSSEY Infrared Imaging System (Li-Cor Bioscience) was used to detect signals according to the manufacturer’s manual.

### Intra-NaccSh injection of *tert*-butyl hydroperoxide

To determine whether the effects of MEGR on nicotine sensitization were mediated by accumbal ROS, the ROS donor *tert-*butyl hydroperoxide (*t*-BOOH; 3.0 μg/200 nL/side; Sigma-Aldrich) [28] was dissolved in MRS and bilaterally administered into the NaccSh via the injectors (internal cannulae, 17 mm; 28-gauge) using motorized syringe pumps over a period of 60 s approximately 60 min after the fourth MEGR treatment. For the intra-NaccSh infusions of *t*-BOOH, stainless steel guide cannulae (15 mm; 23-gauge, 2 mm shorter than the internal cannulae) were bilaterally implanted into the brain using a stereotaxic instrument while the rats were under anesthesia; the cannula tips were placed 2 mm above the NaccSh so that the injectors exactly targeted the NaccSh. Five minutes after *t*-BOOH administration, the rats were systemically challenged with nicotine and tested in the locomotor testing boxes for 60 min. Immediately after the behavioral test, the rats were decapitated and their brains were removed to verify the guide cannula placement.

### Statistical analysis

All data are expressed as mean ± standard error of the mean (SEM). The behavioral and biochemical data (including Western blot analyses) were assessed with one-way analysis of variance (ANOVA) tests followed by Newman–Keuls post-hoc tests. The neurochemical data (in vivo microdialysis) were assessed with a two-way ANOVA followed by Bonferroni post-hoc tests. All tests were conducted with the commercially available software GraphPad Prism 5.0 (GraphPad Software, San Diego, CA, USA) and *p* values <0.05 were considered to indicate statistical significance. The normality of data was checked and the homogeneity of variances was analyzed by Bartlett’s test before each running of ANOVA analyses.

## Results

### Effects of MEGR on nicotine-induced Locomotor sensitization

A nicotine (or saline) challenge was performed on the fourth day after the final daily administration of the repeated nicotine protocol. The RNT rats challenged with nicotine (Nicotine/DW/Nicotine) exhibited a significantly greater increase in locomotor activity than the repeated saline-treated rats (Saline/DW/Saline) and rats that received only a challenge dose of nicotine (Saline/DW/Nicotine) [F_(5, 36)_ = 12.46, *p* < 0.0001; Nicotine/DW/Nicotine group (*n* = 7) vs. Saline/DW/Saline group (*n* = 7), *p* < 0.001; Nicotine/DW/Nicotine group vs. Saline/DW/Nicotine group (*n* = 7), *p* < 0.01]. These findings indicate that RNT induced locomotor sensitization. However, the nicotine-induced behavioral sensitization was attenuated by both doses (60 and 180 mg/kg/d) of MEGR [Nicotine/DW/Nicotine group vs. Nicotine/MEGR60/Nicotine group (*n* = 7), *p* < 0.01; Nicotine/DW/Nicotine group vs. Nicotine/MEGR180/Nicotine group (*n* = 7), *p* < 0.001] in a dose-dependent manner (Nicotine/MEGR60/Nicotine group vs. Nicotine/MEGR180/Nicotine group, *p* < 0.05). Locomotor activity was not significantly altered by acute nicotine treatment (Saline/DW/Nicotine group vs. Saline/DW/Saline group, *p* > 0.05) or treatment with 180 mg/kg/d MEGR (Saline/DW/Saline group vs. Saline/MEGR180/Nicotine group, *p* > 0.05) (Fig. 1b).

### Effects of MEGR on nicotine-sensitized increases in Accumbal DA release

The rats with surgery did not show any significant local infection and physical abnormalities, and there were no significant differences in basal accumbal DA levels among the repeated saline-treated group (Saline/DW/Saline: 3.92 ± 0.45 nM, *n* = 5), the acute nicotine-treated group (Saline/DW/Nicotine: 4.23 ± 0.48 nM, *n* = 5), the RNT group (Nicotine/DW/Nicotine: 3.79 ± 0.38 nM, *n* = 6), the repeated nicotine plus 180 mg/kg/d MEGR group (Nicotine/MEGR180/Nicotine: 4.01 ± 0.43 nM, *n* = 6), and the acute nicotine plus 180 mg/kg/d MEGR group (Saline/MEGR180/Nicotine: 4.32 ± 0.52 nM, *n* = 5). A two-way ANOVA and post-hoc tests revealed that the acute local infusion of nicotine directly into the NaccSh produced much higher elevations of accumbal DA overflow (relative to baseline) in nicotine-pretreated rats than in saline-pretreated rats [F_(treatment × time)_ = 17.35, *p* < 0.0001; F_(treatment)_ = 58.64, *p* < 0.0001; F _(time)_ = 78.32, *p* < 0.0001]. Additionally, significant elevations in accumbal DA were seen at 20 min [Nicotine/DW/Nicotine group (343.24 ± 36.47%) vs. Saline/DW/Saline group, *p* < 0.001; Nicotine/DW/Nicotine group vs. Saline/DW/Nicotine group (201.39 ± 21.04%), *p* < 0.001], 40 min [Nicotine/DW/Nicotine group (700.92 ± 69.35%) vs. Saline/DW/Saline group, *p* < 0.001; Nicotine/DW/Nicotine group vs. Saline/DW/Nicotine group (313.71 ± 30.64%), *p* < 0.001], and 60 min [Nicotine/DW/Nicotine group (286.96 ± 30.11%) vs. Saline/DW/Saline group, *p* < 0.001; Nicotine/DW/Nicotine group vs. Saline/DW/Nicotine group (130.90 ± 14.31%), *p* < 0.001] after a nicotine challenge.

**Fig. 2:**
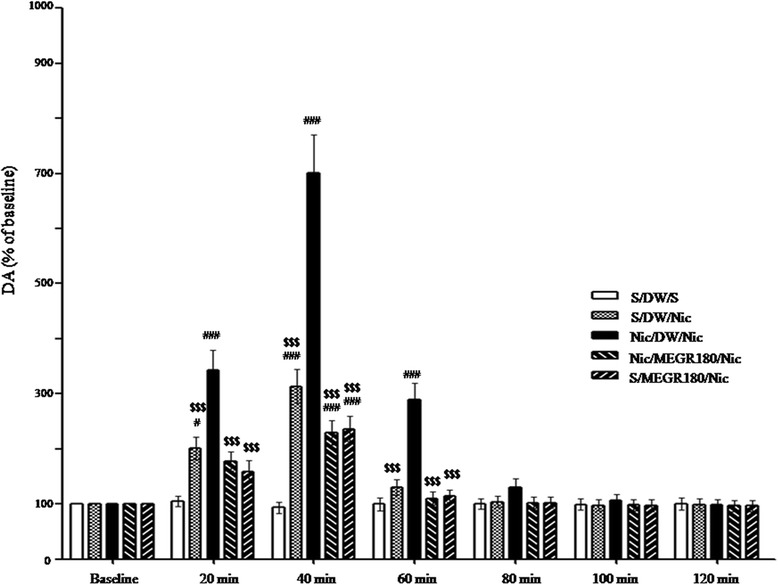
Effects of MEGR on extracellular DA levels in the NaccSh. On the 4th after the last nicotine (Nic) injection, rats were challenged with a direct intra-NaccSh infusion of 3.0 mM Nic, and during the withdrawal period the rats were treated with MEGR. All data are expressed as means ± SEM (*n* = 5–6). Nic: nicotine, S: saline, MEGR: methanol extract of G. *radix*, MEGR180: 180 mg/kg/day MEGR once a day for 4 consecutive days. ^#^ *P* < 0.05, ^###^ *P* < 0.001 versus S/DW/S group; ^$$$^ *P* < 0.001 versus Nic/DW/Nic group (two-way ANOVA followed by the Bonferroni post-hoc test)

However, similar to the locomotor activity results, treatment with MEGR effectively attenuated the sensitized increases in extracellular DA release at 20 min (Nicotine/DW/Nicotine group vs. Nicotine/MEGR180/Nicotine group, *p* < 0.001), 40 min (Nicotine/DW/Nicotine group vs. Nicotine/MEGR180/Nicotine group, *p* < 0.001) and 60 min (Nicotine/DW/Nicotine group vs. Nicotine/MEGR180/Nicotine group, *p* < 0.001). In contrast, post-hoc tests revealed that, unlike the behavioral data, an acute nicotine infusion into the NaccSh increased accumbal DA release at 20 min (Saline/DW/Nicotine group vs. Saline/DW/Saline group, *p* < 0.05) and 40 min (Saline/DW/Nicotine group vs. Saline/DW/Saline group, *p* < 0.001); there was a trend for 180 mg/kg MEGR to inhibit this increase but the difference was not statistically significant (Fig. 2).

### Effects of MEGR on antioxidant profiles in the NaccSh

**Fig. 3:**
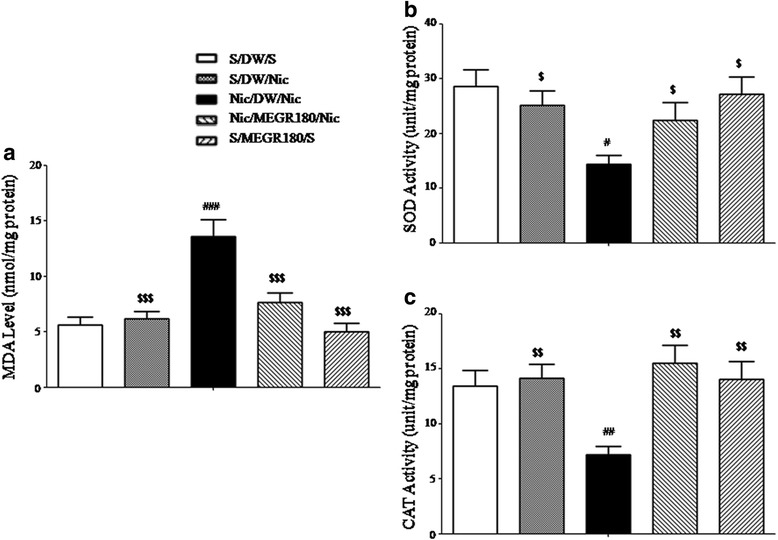
Effects of MEGR on lipid peroxidation and antioxidant enzymes activities in the NaccSh. Immediately after the behavioral test, the **a** MDA levels and the activities of **b** SOD and **c** CAT in the NaccSh of the rats were measured using commercially available kits. All data are expressed as means ± SEM (*n* = 4–5). Nic: nicotine, S: saline, MEGR: methanol extract of G. *radix*, MEGR180: 180 mg/kg/day MEGR once a day for 4 consecutive days. ^#^ *P* < 0.05, ^##^ *P* < 0.01, ^###^ *P* < 0.001 versus S/DW/S group; ^$^ *P* < 0.05, ^$$^ *P* < 0.01, ^$$$^ *P* < 0.001 versus Nic/DW/Nic group (one-way ANOVA followed by Newman-Keuls post-hoc test)

There were significant increases in accumbal MDA production in the nicotine-sensitized rats (Nicotine/DW/Nicotine) compared with the repeated saline-treated rats [F_(4, 18)_ = 12.31, *p* < 0.0001; Nicotine/DW/Nicotine group (*n* = 5) vs. Saline/DW/Saline group (*n* = 4), *p* < 0.001], indicating nicotine sensitization is associated with increased accumbal oxidative stress; however, treatment with 180 mg/kg/d MEGR once a day for 4 d significantly inhibited these increases [Nicotine/DW/Nicotine group vs. Nicotine/MEGR180/Nicotine group (*n* = 5), *p* < 0.001] (Fig. 3a). Accumbal SOD and CAT activities in the nicotine-sensitized group were lower than those in the repeated saline-treated group [SOD: F_(4, 18)_ = 4.18, *p* < 0.05; Nicotine/DW/Nicotine group (*n* = 5) vs. Saline/DW/Saline group (*n* = 4), *p* < 0.05; CAT: F_(4, 18)_ = 6.27, *p* < 0.01; Nicotine/DW/Nicotine group (*n* = 5) vs. Saline/DW/Saline group (*n* = 4), *p* < 0.01]. The alterations of SOD and CAT activities were also ameliorated by treatment with MEGR [SOD: Nicotine/DW/Nicotine group vs. Nicotine/MEGR180/Nicotine (*n* = 5), *p* < 0.05; CAT: Nicotine/DW/Nicotine group vs. Nicotine/MEGR180/Nicotine group (*n* = 5), *p* < 0.01] (Fig. 3b and c). However, neither acute nicotine treatment nor MEGR administration alone significantly changed the oxidant/antioxidant state in the NaccSh [MDA, SOD, and CAT: Saline/DW/Nicotine group (*n* = 5) vs. Saline/DW/Saline group, *p* > 0.05; Saline/MEGR180/Saline group (*n* = 4) vs. Saline/DW/Saline group, *p* > 0.05].

### Effects of MEGR on Nrf2 and HO-1 protein expression in the NaccSh

**Fig. 4:**
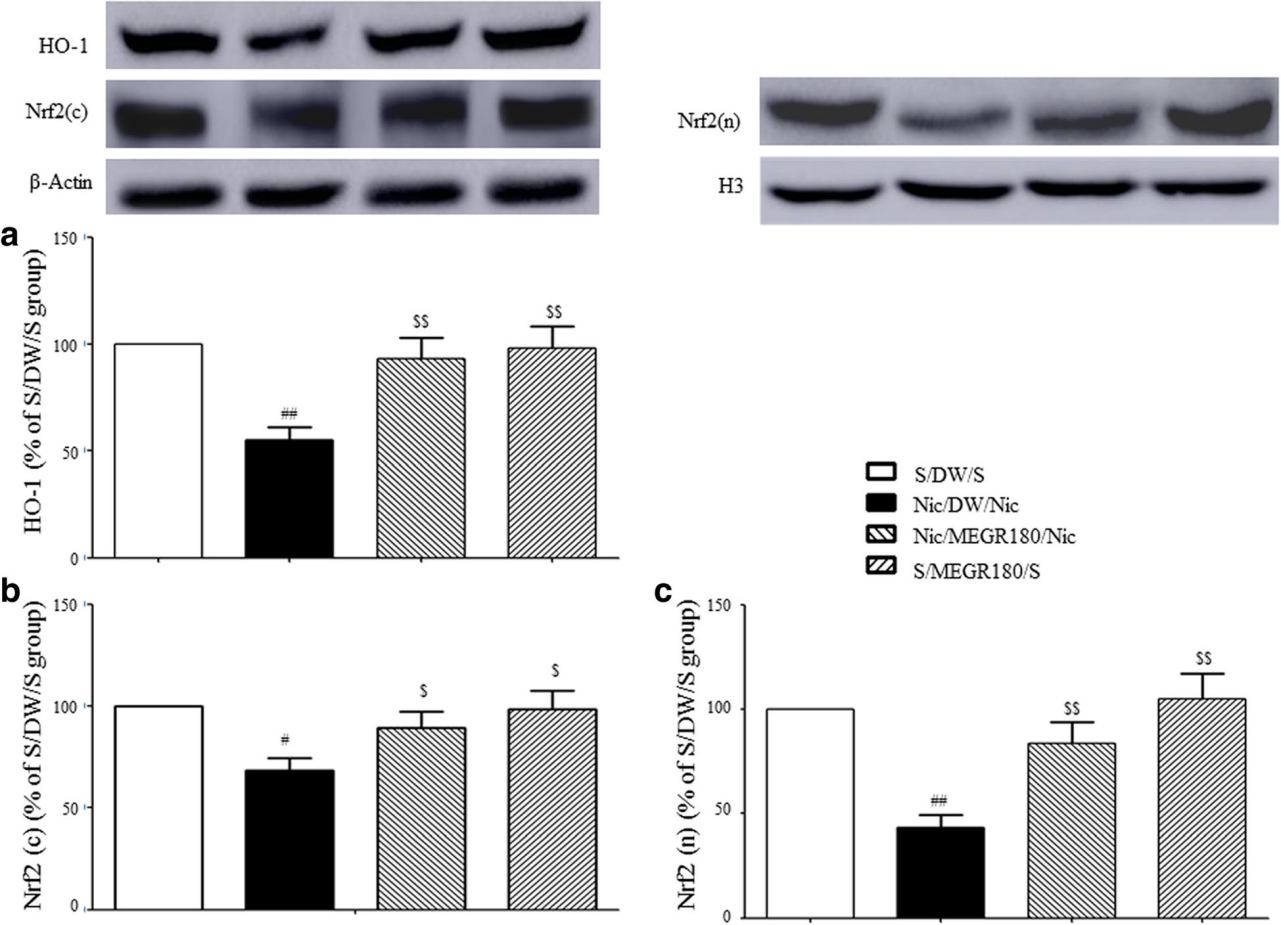
Effect of MEGR on the Nrf2 and HO-1 expressions in the nicotine-sensitized NaccSh. Immediately after the behavioral test, the protein levels of **a** cytoplasmic HO-1 and **b** cytoplasmic and **c** nuclear Nrf2 in the NaccSh were evaluated by Western blot assays. All data are expressed as means ± SEM (*n* = 4). Nic: nicotine, S: saline, H3: Histone H3, S: saline, DW: distilled water, MEGR: methanol extract of *G. radix*, MEGR180: 180 mg/kg/day MEGR. ^#^ *P* < 0.05, ^##^ *P* < 0.01 versus S/DW/S group; ^$^ *P* < 0.05, ^$$^ *P* < 0.01 versus Nic/DW/Nic group (one-way ANOVA followed by Newman-Keuls post-hoc test)

The protein expressions of cytoplasmic Nrf2 and HO-1 in the NaccSh exhibited a reduction in the nicotine-sensitized group compared with those in the repeated saline-treated group [Nrf2: F_(3, 12)_ = 4.64, *p* < 0.05; Nicotine/DW/Nicotine group (*n* = 4) vs. Saline/DW/Saline group (*n* = 4), *p* < 0.05; HO-1: F_(3, 12)_ = 7.37, *p* < 0.01; Nicotine/DW/Nicotine group (*n* = 4) vs. Saline/DW/Saline group (*n* = 4), *p* < 0.01]. Additionally, the nuclear protein expressions of Nrf2 in the NaccSh were lower in the nicotine-sensitized rats than in the repeated saline-treated rats [F_(3, 12)_ = 10.71, *p* < 0.01; Nicotine/DW/Nicotine group (*n* = 4) vs. Saline/DW/Saline group (*n* = 4), *p* < 0.01]. However, these downregulated protein expressions were reversed by treatment with 180 mg/kg/d MEGR once a day for 4 d during the nicotine withdrawal period [Nrf2 (cytoplasmic): Nicotine/DW/Nicotine group vs. Nicotine/MEGR180/Nicotine group (*n* = 4), *p* < 0.05; HO-1: Nicotine/DW/Nicotine group vs. Nicotine/MEGR180/Nicotine group (*n* = 4), *p* < 0.01; Nrf2 (nuclear): Nicotine/DW/Nicotine group vs. Nicotine/MEGR180/Nicotine group (*n* = 4), *p* < 0.01]. MEGR treatment alone did not produce significant changes in the cytoplasmic and nuclear protein levels of Nrf-2 or HO-1 [Nrf2 (cytoplasmic), HO-1, and Nrf2 (nuclear): Saline/MEGR180/Saline group (*n* = 4) vs. Saline/DW/Saline group, *p* > 0.05] (Fig. 4).

### Effects of intra-NaccSh infusion of *t*-BOOH on the anti-sensitization action of MEGR

**Fig. 5:**
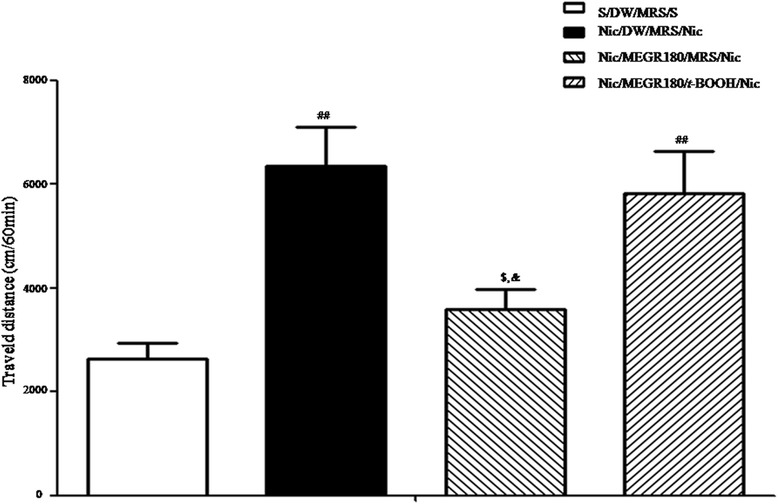
Effects of intra-NaccSh infusion of *t*-BOOH on the anti-sensitization action of MEGR. Sixty minutes after the 4th MEGR, rats received bilateral infusions of *t*-BOOH into the NaccSh, were systemically challenged with nicotine, and then tested in the locomotor testing boxes for 60 min. All data are expressed as means ± SEM (*n* = 4–5). Nic: nicotine, S: saline, MRS: modified Ringers’ solution, MEGR: methanol extract of G. *radix*, MEGR180: 180 mg/kg/day MEGR once a day for 4 consecutive days. ^##^ *P* < 0.01versus S/DW/MRS/S group; ^$^ *P* < 0.05 versus Nic/DW/MRS/Nic group; ^&^ *P* < 0.05 versus Nic/MEGR60/*t*-BooH/Nic group (one-way ANOVA followed by Newman-Keuls post-hoc test)

Consistent with the locomotor activity findings, RNT sensitized the locomotor response to a challenge dose of nicotine [F_(3, 14)_ = 8.37, *p* < 0.01; Nicotine/DW/MRS/Nicotine group (*n* = 4) vs. Saline/DW/MRS/Saline group (*n* = 4), *p* < 0.01] and 180 mg/kg/d MEGR prevented the occurrence of this behavioral sensitization [Nicotine/DW/MRS/Nicotine group vs. Nicotine/MEGR180/MRS/Nicotine group (*n* = 5), *p* < 0.05]. However, a post-MEGR infusion of *t*-BOOH into the NaccSh abolished the ability of MEGR to prevent sensitization [Nicotine/MEGR180/MRS/Nicotine group vs. Nicotine/MEGR180/*t*-BOOH/Nicotine group (*n* = 5), *p* < 0.05] (Fig. 5).

## Discussion

In the present study, RNT-induced locomotor sensitization was dose-dependently inhibited by MEGR (60 or 180 mg/kg/d once a day for 4 d) such that the 180 mg/kg/d dose almost completely abolished the sensitization. Additionally, 180 mg/kg/d MEGR blocked the sensitization-induced increase in DA release in the NaccSh. The present study also showed that there was an increase in MDA production, reduced SOD and CAT activities, and decreases in the expression of Nrf2 and HO-1 in the nicotine-sensitized NaccSh; these changes were all ameliorated by 180 mg/kg/d MEGR. These results suggest that MEGR exerted inhibitory effects on nicotine-induced locomotor activity and accumbal dopaminergic sensitization via its antioxidant action.

In the present study, the repeated systemic administration of nicotine induced behavioral sensitization as evidenced by the increased distance traveled by nicotine-pretreated rats than by saline control rats when re-exposed to the same dose of nicotine [6, 26]. This behavioral sensitization was blocked by both doses of MEGR (60 and 180 mg/kg/d), which is consistent with previous findings from our research group showing that these same doses of MEGR attenuated repeated methamphetamine-induced locomotor sensitization [21]. These results indicate that MEGR has an inhibitory effect on nicotine-induced locomotor sensitization. Locomotor sensitization is a behavioral phenotype associated with a sensitized mesoaccumbal dopaminergic response, which is also supported by previous findings from our group [6, 26]. In the present study, a challenge infusion of nicotine into the NaccSh elicited significantly greater accumbal DA release in nicotine-pretreated rats than in saline control rats. However, this increase was effectively abolished by 180 mg/kg/d MEGR. These results provide neurochemical support for the inhibitory effects of MEGR on RNT-induced behavioral sensitization and imply that the behavioral effects are mediated by the ability of MEGR to mask the sensitized accumbal DA response.

Nicotine binds to nicotinic acetylcholine receptors (nAchRs) to induce neurotransmitter release, including accumbal DA, in the brain [29]. This action is physiologically designated as neuronal signal transmission as well as biochemically denoted by an altered cellular energy state coupled with the production of ROS [30]. RNT alters activity within the tegmental-accumbal dopaminergic system that underlies behavioral and accumbal dopaminergic sensitization, which is biochemically sustained by heightened accumbal ROS levels. RNT increases the production of lipid hydroperoxides in the cerebral cortex and hippocampus of rats [31]. RNT-induced behavioral sensitization and conditioned place preference in mice occur in conjunction with the dysregulation of accumbal membrane metabolism and an increase in hippocampal MDA production [32, 33]. Our group has previously reported that rats self-administering cocaine and methamphetamine show an enhanced production of accumbal ROS, which is associated with sensitized accumbal DA release [9, 10]. The present study found RNT-induced elevation in MDA levels and decreases in SOD and CAT activities in the NaccSh, which supports the finding that repeated psychostimulant treatment increases accumbal oxidative stress. The present study also found that 180 mg/kg/d MEGR prevented increases in accumbal oxidative stress and that the post-MEGR administration of *t*-BOOH abolished the inhibitory effects of MEGR on nicotine-induced locomotor sensitization. These results indicate that MEGR blocks RNT-induced sensitization by counteracting increases in accumbal oxidative stress.

Eukaryotic cells have a fine-tuned oxidant/antioxidant system that maintains normal cellular functioning. Nrf2 is a basic leucine zipper transcription factor that plays a central role in the antioxidant process. Under normal conditions, it resides in the cytoplasm and is quickly degraded by a cluster of proteins, including Keap1 and Cullin3. However, once stressful events occur, Nrf2 is disassociated from Keap1 and translocated into the nucleus to bind to DNA promoters where it eventually initiates the transcription of several antioxidant and phase II detoxifying enzymes, including HO-1 [34], thus protecting cells against injury.

In the present study, RNT reduced Nrf2 protein expression in both the cytoplasmic and nuclear fractions of the NaccSh, which is consistent with studies showing that beta-amyloid-induced stress decreased hippocampal Nrf2 expression [35] and that traumatic brain injury disrupted the nuclear translocation of Nrf2 in the cerebral cortex [36]. The present study also found decreased HO-1 expression in the nicotine-sensitized NaccSh. Taken together, these results indicate that RNT elicits dysregulation in the Nrf2–HO-1 pathway and that the disturbance in cellular antioxidant function underlies the enhancement of accumbal oxidative stress which, in turn, further mediates RNT-induced behavioral and neurochemical sensitization. On the other hand, the present study also showed that 180 mg/kg/d MEGR markedly increased the expression of Nrf2 and HO-1 in the nicotine-sensitized NaccSh but spared it in the nicotine-free NaccSh. Taken together, these findings suggest that the effects of MEGR on RNT-induced sensitization and accumbal oxidative stress are due to improvements in altered cellular antioxidant systems, such as the Nrf2–HO-1 pathway.

## Conclusions

In the present study, 60 and 180 mg/kg/d MEGR dose-dependently suppressed RNT-induced locomotor sensitization and 180 mg/kg/d MEGR reduced sensitized accumbal DA release. Additionally, MEGR attenuated RNT-induced increases in MDA levels and decreases in SOD and CAT activities in the NaccSh and rescued downregulated function in the Nrf2–HO-1 pathway. These data suggest that *G.* radix can block RNT-induced behavioral and neurochemical sensitization by counteracting accumbal oxidative stress, and further provide the possibility that *G.* radix can be an important herbal source to develop new agents to aid smoking cessation.

## Abbreviations

CAT: Catalase
DA: Dopamine
DW: Distilled water
*G.* radix: *Glycyrrhizae* radix
HO-1: Heme oxygenase 1
HPLC: High-performance liquid chromatography
MDA: Malondialdehyde
MEGR: Methanol extract from *G.* radix
MEGR60 (180): 60 (180) mg/kg/d MEGR
MRS: Modified Ringer’s solution
Nacc: Nucleus accumbens
NaccSh: Nacc shell
Nrf2: Nuclear factor erythroid 2–related factor 2
RNT: Repeated nicotine treatment
ROS: Reactive oxygen species
SOD: Superoxide dismutase
*t*-BOOH: *tert*-butyl hydroperoxide
